# Quantities and units for centrifugation in the clinical laboratory

**DOI:** 10.1155/S1463924692000208

**Published:** 1992

**Authors:** M. Lauritzen

**Affiliations:** Novo Nordisk A/S Produktionsvej 8 Glostrup DK-2600 Denmark

## Abstract

The centrifuge is widely used in clinical laboratories for the separation of components. For example in laboratories performing biochemical analyses on body fluids it is routinely used to separate blood cells from plasma, to separate sediment from urine, to measure the volume fraction of erythrocytes in blood (the haematocrit), and to separate bound from free components in protein binding and immunoprocedures. Less routinely, centrifugation is used for separation of lipoproteins in reference procedures for their measurement, separation of cellular components, and separation of DNA fragments. Various quantities are used for the description and the calculation of the separation processes at centrifugation. The aim of this document is to provide manufacturers and users of centrifuges with a list of quantities and units for centrifugation consistent with the International System of Units, SI, and standards of the International Organization for Standardization (ISO).


Journal of Automatic Chemistry, Vol. 14, No. 3 (May–June 1992), pp. 93–96

### Quantities and units for centrifugation in the clinical laboratory

**M. Lauritzen for the International Union of Pure and Applied Chemistry, Clinical Chemistry Division, Commission VII.2: Quantities and Units in Clinical Chemistry and the International Federation of Clinical Chemistry* Scientific Division Committee on Quantities and Units**

*The centrifuge is widely used in clinical laboratories for the separation of components. For example in laboratories performing biochemical analyses on body fluids it is routinely used to separate blood cells from plasma, to separate sediment from urine, to measure the volume fraction of erythrocytes in blood (the haematocrit), and to separate bound from free components in protein binding and immunoprocedures. Less routinely, centrifugation is used for separation of lipoproteins in reference procedures for their measurement, separation of cellular components, and separation of DNA fragments. Various quantities are used for the description and the calculation of the separation processes at centrifugation. The aim of this document is to provide manufacturers and users of centrifuges with a list of quantities and units for centrifugation consistent with the International System of Units, SI, and standards of the International Organization for Standardization (ISO).*

### General definitions of quantities and units

*Quantity*

A quantity is a measurable property, physical or chemical, of a specified system. It can be expressed as a product of a numerical value and a unit:

quantity = numerical value · unit.

*Base kind-of-quantities and base units*

By convention of the International System of Units (SI), quantities are organized in a dimensional system built upon seven *base kind-of-quantities*, each of which is regarded as having its own dimension and is considered to be dimensionally independent of the other base kind-of-quantities. For each base kind-of-quantity, a *base unit* is defined.

*Derived kind-of-quantities and derived units*

All other quantities are *derived-kind-of-quantities* defined algebraically from base-kind-of-quantities. *Derived units* are defined analogously.

* Comments on this paper and requests for offprints should be sent to Dr M. Lauritzen at Novo Nordisk A/S, Produktionsvej 8, DK-2600 Glostrup, Denmark.

*Table 1. Base kind-of-quantities and SI base units.**

| Quantity | | SI unit | |
|---|---|---|---|
| Name | Symbol | Name | Symbol |
| Length | $l$ | Metre | m |
| Mass | $m$ | Kilogram | kg |
| Time | $t$ | Second | s |
| Electric current | $I$ | Ampere | A |
| Thermodynamic temperature | $T$ | Kelvin | K |
| Amount of substance | $n$ | Mole | mol |
| Luminous intensity | $I_v$ | Candela | cd |

* The symbols for the kind-of-quantities are recommended symbols.

### Alphabetic list of kind-of-quantities and units for centrifugation

| *Kind-of-quantity* **Name,** *synonym(s), definition, comment(s)* | *Symbol* | *Coherent SI unit* *Symbol* |
|---|---|---|
| **Acceleration** *Definition:* Rate of change of velocity: $a = dv/dt$. *Comment:* Acceleration is a vector quantity. | $a$ | $m\ s^{-2}$ |
| **Acceleration of free fall** *Synonym:* Acceleration due to gravity. *Definition:* Acceleration at a free fall *in vacuo* due to gravity. *Comment:* Acceleration of free fall is a vector quantity. | $g$ | $m\ s^{-2}$ |
| **Avogadro constant** *Definition:* Number of entities in a system divided by the amount of substance of these entities: $L = N/n = 6{\cdot}022\ 136\ 7 \cdot 10^{23}$ $mol^{-1}$. | $L$, $N_A$ | $mol^{-1}$ |
| **Boltzmann constant** *Synonym:* Molecular gas constant or entitic gas constant. *Definition:* Molar gas constant divided by the Avogadro constant: $k = R/L = 1{\cdot}380\ 658 \cdot 10^{-23}$ $J\ K^{-1}$. | $k$, $k_B$ | $J\ K^{-1}$ |

© 1992 IFCC

**Centrifugal acceleration** $a_{\mathrm{rot}}$ m s$^{-2}$
*Definition:* Acceleration of a component as a result of a uniform rotational motion.
*Comment:* Centrifugal acceleration is a vector quantity.

**Centrifugal force** $F_{\mathrm{rot}}$ N = m kg s$^{-2}$
*Definition:* Force acting on a body as a result of centrifugal acceleration:
$F_{\mathrm{rot}} = m\, a_{\mathrm{rot}}$.
*Comment:* Centrifugal force is a vector quantity.
The centrifugal force equals the product of mass and the centrifugal acceleration of the body.

**Centrifugal radius** $r$ m
*Definition:* Radius at which a component is spinning at the end of the period of centrifugation.
*Comment:* For a component sedimented from a dilute suspension, it can be equated with radius of rotation at the bottom of the centrifuge tube.

**Circular frequency** $\omega$ s$^{-1}$ = rad s$^{-1}$

*Synonym:* Angular frequency.

*Definition:* 2π times the frequency:

$\omega = 2\pi f$.

**Diffusion coefficient** (of component B) $D_{\mathrm{B}}$ m$^2$ s$^{-1}$
*Definition:* Absolute value of the product of local number concentration of the component and local average velocity of particles of that component divided by number concentration gradient in the direction of movement:
$D_{\mathrm{B}} = |\, C_{\mathrm{B}}\, v_{\mathrm{B}} \,| / \mathrm{grad}\, C_{\mathrm{B}}$.

**Force** (acting on a body) $F$ N = kg m s$^{-2}$
*Definition:* Product of the mass of a body and its acceleration:
$F = m\, a$.
*Comment:* Force is a vector quantity.
The resultant force acting on a body is equal to the rate of change of momentum of the body.

**Kinetic energy** (of a body in uniform motion) $E_{\mathrm{k}}$ J = kg m$^2$ s$^{-2}$
*Definition:* Half of the product of mass and square of velocity of the body:
$E_{\mathrm{k}} = \frac{1}{2} m\, v^2$.

**Mass concentration** (of component B) $\gamma_{\mathrm{B}}$, $\rho_{\mathrm{B}}$ kg m$^{-3}$
*Definition:* Mass of the component divided by the volume of the system:
$\gamma_{\mathrm{B}} = m_{\mathrm{B}}/V$.

**Mass density** $\rho$ kg m$^{-3}$
*Synonym:* Volumic mass.
*Definition:* Mass of the system divided by its volume:
$\rho = m/V$.

**Mass density gradient** grad ρ kg m$^{-4}$
*Synonym:* Volumic mass gradient.
*Definition:* Differential change of mass density with distance in direction $x$:
$\mathrm{grad}_x\, \rho = \mathrm{d}\rho/\mathrm{d}x$.
*Comment:* Colloidal components may be fractionated by centrifugation in a fluid with a gradient obtained by a suitable solute, for instance potassium bromide in water. Mass density gradient is a vector quantity.

**Mass fraction** (of component B) $w_{\mathrm{B}}$ 1
*Definition:* Mass of the component divided by mass of all components in the system:
$w_{\mathrm{B}} = m_{\mathrm{B}}/\Sigma m_i$.

**Molar gas constant** $R$ J K$^{-1}$ mol$^{-1}$
*Definition:* Universal constant of proportionality in the ideal gas law:
$p\, V_{\mathrm{m}} = R\, T$
$R = 8{\cdot}314\,511$ J K$^{-1}$ mol$^{-1}$.
*Comment:* The gas constant equals the product of the Avogadro constant and the Boltzmann constant.

**Molar mass** (of component B) $M_{\mathrm{B}}$ kg mol$^{-1}$

*Definition:* Mass of the component divided by its amount of substance:

$M_{\mathrm{B}} = m/n_{\mathrm{B}}$.

**Molar volume** (of component B) $V_{\mathrm{m,b}}$ m$^3$ mol$^{-1}$
*Definition:* Volume of the component divided by its amount of substance:
$V_{\mathrm{m,b}} = V/n_{\mathrm{B}}$.

**Moment of inertia** (of body about an axis) $I$ kg m$^2$
*Synonym:* Dynamic moment of inertia.
*Definition:* Sum (or integral) of the products of the mass elements of a body and the squares of their respective distances from the axis:
$I = \Sigma\, m_i r_i^2$.

**Number concentration** (of component B) — $C_B$ — $m^{-3}$
*Definition:* Number of entities of stated type for that component divided by the volume of the system:
$C_B = N_B/V$.
*Comment:* Besides molecules or particles, the type of entity may, for instance, be a chemical group within molecules or an ionic charge, and is therefore broader than the concepts 'molecular concentration' and 'particle concentration'.

**Number concentration gradient** (of component B) — grad $C_B$ — $m^{-4}$
*Definition:* Differential change of number concentration of component B with distance in direction $x$:
$\mathrm{grad}_x\, C_B = \mathrm{d}C_B/\mathrm{d}x$.
*Comment:* Number concentration gradient is a vector quantity.

**Partial mass density** (of component B) — $\rho_B$ — kg $m^{-3}$
*Synonym:* Partial volumic mass.
*Definition:* Change in mass of the component due to addition of a differentially small amount of that component, divided by the change in volume of the system:
$\rho_B = \mathrm{d}m_B/\mathrm{d}V$.

**Partial specific volume** (of component B) — $v_B$ — $m^3\ kg^{-1}$
*Synonym:* Partial massic volume.
*Definition:* Change in volume of a system when a differentially small amount of a component is added, divided by the mass of that component:
$v_B = \mathrm{d}V/\mathrm{d}m_B$.
*Comment:* Partial specific volume is used in estimation of molar mass of colloidal particles (for example viruses or nucleic acids) from the sedimentation coefficient.

**Pressure** — $p$ — Pa $= N\ m^{-2}$ $= kg\ s^{-2}\ m^{-1}$

*Definition:* Force divided by area:

$p = F/A$.

**Rate coefficient** (of a suspended component B in a fluid) — $k_B$ — $s^{-1}$
*Definition:* Number fraction of particles of the component passing a given position in the direction of gravitational or centrifugal acceleration, divided by time of passage:
$k_B = -\mathrm{d}N_B/(N_B\,\mathrm{d}t) = -(\mathrm{dln}\ N_B)/\mathrm{d}t$.

**Rotational frequency** — $f_{rot}$ — Hz $= s^{-1}$
*Definition:* Number of rotations divided by time:
$f_{rot} = \mathrm{d}N/\mathrm{d}t$.
*Comment:* The synonyms: rate of rotation, rate of revolution, centrifugal speed, centrifugation speed, and the traditional units of rotational frequency such as revolutions per minute, r.p.m., rpm, rev./min, r/min, are not recommended.

**Sedimentation coefficient** (of a suspended component B in a fluid) — $s_B$ — s
*Definition:* Reciprocal of the rate coefficient of the component passing a given position in the direction of gravitational or centrifugal acceleration:
$s_B = (k_B)^{-1} = -N_B\,\mathrm{d}t/\mathrm{d}N_B = -\mathrm{d}t/\mathrm{dln}\ N_B$.
*Comment:* Use of the 'Svedberg unit', Sv $= 10^{-13}$ s, is not recommended.
In current usage, subscripts are added to the symbol to indicate temperature and medium, and superscripts to indicate concentration.

**Sedimentation velocity** (of a suspended component B in a fluid) — $v_B$ — $m\ s^{-1}$
*Definition:* Velocity of the component relative to the fluid in the direction of gravitational or centrifugal acceleration:
$v_B = \mathrm{d}l_B/\mathrm{d}t$.
*Comment:* Sedimentation velocity is a vector quantity.

**Specific volume** — $v$ — $m^3\ kg^{-1}$
*Synonym:* Massic volume.
*Definition:* Volume of a system divided by its mass:
$v = V/m = \rho^{-1}$.
*Comment:* Specific volume is the reciprocal of mass density.

**Standard acceleration of free fall** — $g_n$ — $m\ s^{-2}$
*Definition:* Acceleration of free fall at sea level for the latitude 45°:
$g_n = 9{\cdot}806\,65\ m\ s^{-2}$ exactly.

**Substance concentration** (of component B) — $c_B$ — mol $m^{-3}$
*Definition:* Amount of substance of the component divided by the volume of the system:
$c_B = n_B/V$.

**Time of centrifugation** $t$ s
*Definition:* Time difference from switching on until switching off.
*Comment:* The time for deceleration is not included.

**Velocity** $v$ m s$^{-1}$
*Definition:* Distance travelled divided by time of travel:
$v = \mathrm{d}l/\mathrm{d}t$.
*Comment:* Velocity is a vector quantity.

**Viscosity** $\eta$ Pa s
*Synonym:* Dynamic viscosity.
*Definition:* Constant of proportionality for shear stress, $\tau_{xz}$, in a fluid moving with a velocity gradient, $\mathrm{d}v_x/\mathrm{d}z$, perpendicular to the plane of shear:
$\tau_{xz} = \eta\, \mathrm{d}v_x/\mathrm{d}z$.
*Comment:* This definition applies to laminar flow for which $v_z = 0$.

**Volume** $V$ m$^3$, (l), L
*Comment:* The unit litre, L = 0·001 m$^3$, is customarily used in clinical laboratories instead of m$^3$ for reporting of analytical results and is recognized for use with SI.

### Appendix

*Examples of calculation*

**Centrifugal acceleration, $a_{rot}$**
The centrifugal acceleration may be calculated from a stated radius and the rotational frequency:
$a_{rot} = 4\pi^2\, r f_{rot}^2$.
Centrifugal acceleration is commonly expressed in terms of standard acceleration $g_n$:
1 m s$^{-2}$ = (1/9·806 65) $g_n$
$a_{rot} = (4\pi^2/9{\cdot}806\,65)(r/\mathrm{m})(f_{rot}/\mathrm{Hz})^2\, g_n$.
*Example:*
Radius at which the component is spinning at the end of centrifuging: $r$ = 170 mm.
Rotational frequency: $f_{rot}$ = 50 Hz (= 50 s$^{-1}$ = 3000 min$^{-1}$).
$a_{rot} = (4\pi^2/9{\cdot}807)(170\ \mathrm{mm/m})(50\ \mathrm{Hz/Hz})^2\, g_n$.
$a_{rot} = (4{\cdot}025\,7)(170 \cdot 0{\cdot}001)(50)^2\, g_n = 1711\, g_n$.

**Kinetic energy, $E_k$**
The kinetic energy of a *rotating body* may be calculated by summation of all contributions from partial masses $m_i$ of the body at distances $r_i$ from the axis of rotation:
$E_k = 2\pi^2 f_{rot}^2\, \Sigma(m_i\, r_i^2) = 2\pi^2 f_{rot}^2\, I$.
For a *uniform disc*:
$E_k = \pi^2\, m\, r^2 f_{rot}^2$ ($m$ is total mass).
For a *uniform ring* with outer radius $r$ and inner radius $r_i$:
$E_k = \pi^2\, m\, r^2 f_{rot}^2(1 - (r_i/r)^2)$.

**Molar mass** (of component B), $M_B$
Molar mass of an entity B, sedimentating in a fluid, may be calculated from the 'Svedberg equation':
$M_B = (R\, T\, s_B)/(D_B(1 - v_B\rho))$.

*Example:*

$R$ = 8·315 J K$^{-1}$ mol$^{-1}$ (= 8·135 kg m$^2$ s$^{-2}$ K$^{-1}$ mol$^{-1}$); $T$ = 293 K

$s_B$ = 2·10 · 10$^{-13}$ s (sedimentation coefficient); $D_B$ = 6·72 · 10$^{-11}$ m$^2$ s$^{-1}$ (diffusion coefficient)

$v_B$ = 0·722 L/kg (partial specific volume); $\rho$ = 1·00 kg/L (mass density of fluid)

$M_B$ = (8·315 J K$^{-1}$ mol$^{-1}$ · 293 K · 2·10 · 10$^{-13}$ s)/(6·72 · 10$^{-11}$ m$^2$ s$^{-1}$ (1 − (0·722 L/kg)(1·00 kg/L)))

$M_B$ = 27·4 kg mol$^{-1}$.

### Greek letter symbols

Gamma ($\gamma$) = mass concentration.

Eta ($\eta$) = viscosity.

Rho ($\rho$) = mass density and partial mass density.

Omega ($\omega$) = circular frequency.

### Bibliography

1. International Federation of Clinical Chemistry. Expert Panel on Quantities and Units in Clinical Chemistry. Dybkær, R. Approved recommendations (1978). Quantities and units in clinical chemistry. *Clinica Chimica Acta*, **96** (1979), 155F–183F.
2. International Organization for Standardization. *ISO Standards Handbook 2. Units of Measurement* (ISO Central Secretariat, Geneva, 1982).
3. Bureau International des Poids et Mesures. *Le Système International d'Unités (SI)*, 5th French and English Edition (Sèvres, 1985).
4. International Union of Pure and Applied Chemistry. Physical Chemistry Division. Mills, I., Cvitas, T., Homann, K., Kallay, N., Kuchitsu, K., *Quantities, Units and Symbols in Physical Chemistry* (Blackwell Scientific Publications, Oxford, 1988).

